# Practitioner Review: Treatments for young people who self‐harm – challenges and recommendations for research and clinical practice

**DOI:** 10.1111/jcpp.14052

**Published:** 2024-08-28

**Authors:** Katrina Witt, Anne Stewart, Keith Hawton

**Affiliations:** ^1^ Centre for Youth Mental Health The University of Melbourne Parkville Vic. Australia; ^2^ Orygen Parkville Vic. Australia; ^3^ Department of Psychiatry University of Oxford Oxford UK; ^4^ Department of Psychiatry, Centre for Suicide Research University of Oxford Oxford UK

**Keywords:** Suicide, self‐harm, child, adolescence, treatment, prevention

## Abstract

**Background:**

Self‐harm is very common in young people and is associated with suicide. Rates of both self‐harm and suicide have increased in young people, particularly in females. There is a clear need to identify new approaches to prevent repeat self‐harm.

**Method:**

We significantly update and build on previous reviews with the aim of identifying issues in research relevant to clinical practice. We identify challenges in developing, implementing and evaluating treatments for self‐harm in children and adolescents, suggest a way forward for research, and provide clear and practical guidance for clinicians on how to apply current research evidence in the real world.

**Results:**

Currently, there is limited evidence for effective interventions, other than some support for dialectical behaviour therapy for adolescents (DBT‐A). To improve research and, by extension, clinical practice, future studies need to address psychosocial factors associated with youth self‐harm and suicide, investigate the critical mechanism(s) of action, ensure trials are sufficiently powered and representative, and involve young people more actively in the design, implementation and evaluation of these approaches. Consideration should also be given to alternative research designs, such as pragmatic or adaptive clinical trials, as well as registry‐based randomised controlled trials which leverage administrative data collected in routine clinical practice, to help meet these goals.

**Conclusions:**

Recommendations for practice include undertaking comprehensive assessment and formulation, and offering DBT‐A where indicated. There should be further development and evaluation (with input from young people) of Cognitive Behavioural‐based Therapy adapted for young people. Greater attention to the role of the therapeutic relationship and family involvement (where possible) is also an important considerations, irrespective of the specific therapeutic modality. Finally, more consideration should be given to improving staff training to ensure all clinical staff feel equipped to treat young people who self‐harm in a person‐centred and compassionate manner.

## Introduction

Worldwide, while suicide in children is relatively rare, it is the third leading cause of death in children aged 10–14 years. Rates are generally higher in older adolescents and young adults (Glenn et al., 2020), with suicide being the second leading cause of death among both adolescents (aged 15–19 years) and young adults (aged between 20–24 years) (World Health Organization, 2018). Whilst age‐standardised suicide rates for all ages have been decreasing in recent decades (Naghavi, 2019), suicide rates in young people have increased across a number of countries over the last two decades in both males and females (Glenn et al., 2020).

Non‐fatal self‐harm in young people is very common, frequently repeated and associated with increased risk of suicide (Hawton et al., 2012, 2020). For every young male aged 12–17 years who dies by suicide, there are an estimated 100 episodes of hospital‐presenting self‐harm. For every young female between these ages who dies by suicide, there are around 1,000 episodes of hospital‐presenting self‐harm (Geulayov et al., 2018). Many more young people engage in self‐harm in the community. Self‐harm typically begins in early adolescence, peaking in frequency around age 16 years (Gillies et al., 2018). Rates of non‐fatal self‐harm have also increased over recent decades, both in the community (Gillies et al., 2018; Tørmoen, Myhre, Walby, Grøholt, & Rossow, 2020) and according to the number of episodes resulting in presentation to hospital (Griffin et al., 2018), including after the initial phase of the COVID‐19 pandemic (Wong et al., 2023).

It is important to note that, in this practitioner review, we do not favour the approach commonly used in North America of distinguishing between non‐suicidal self‐injury (NSSI) and attempted suicide. This is because there is a high degree of co‐occurrence between the two, particularly in young people (Andover, Morris, Wren, & Bruzzese, 2012). The self‐reported motivations for self‐harm are also complex (Rodham, Hawton, & Evans, 2004), and can change, even within a single episode (De Beurs, Vancayseele, Van Borkulo, Portzky, & Van Heeringen, 2018). Instead, we take the approach favoured in Europe and most of the rest of the world, defining self‐harm as including any intentional act of self‐injury or self‐poisoning irrespective of motivation or degree of suicidal intent (Hawton et al., 2003).

Whilst some psychosocial interventions may have potential for reducing repeat self‐harm and suicide in young people, the quality of the current evidence‐base is limited, as indicated in our recent Cochrane Review (Witt et al., 2021a). There have been relatively few independent evaluations of these interventions in children and adolescents, particularly when compared with the number of intervention trials in adults who have self‐harmed. Also, most trials were judged to have some risk of bias, with weaknesses most commonly observed with respect to adequacy of blinding of outcome assessors, selection of the reported result and measurement of the primary outcome of repetition of self‐harm. Furthermore, we found that although sample sizes have increased over time, many trials are still underpowered to detect effects on proportions of young people repeating self‐harm (Witt et al., 2021a).

In higher‐income countries, clinical practice guidelines generally recommend that all persons presenting to services after self‐harm should receive a comprehensive psychosocial assessment by mental health clinicians to help identify their needs and also risk/protective factors that can be addressed during treatment. Despite this, a recent international review found that one‐quarter of young people presenting to emergency departments after self‐harm in higher‐income countries do not receive these assessments. Fewer still receive such assessments in lower‐income countries, although currently, only one study from a lower income country has reported data for this outcome in young people (Witt et al., 2024). This represents a missed opportunity to tailor appropriate aftercare to the specific treatment needs of this group.

A related international review further found that of those who do receive a psychosocial assessment, around half are referred to outpatient treatment and just over one‐quarter of those referred attend at least one treatment session (Witt et al., 2023), although it was not always clear what type of therapy was offered and, for some young people who self‐harm, one treatment session may be sufficient. A smaller proportion are referred to psychiatric inpatient treatment. There is considerable variability in these estimates both between and within countries that is not clearly related to availability or capacity within the mental health system (Witt et al., 2023, 2024). Importantly, however, rates of referral and receipt of psychosocial treatment for young people engaging in self‐harm have not meaningfully improved over time (Witt et al., 2023, 2024).

Of those who do receive treatment, adherence rates remain low, particularly for outpatient treatment. Only around one‐third of young people presenting to hospital following an episode of self‐harm will attend all follow‐up outpatient treatment sessions that are arranged for them (Granboulan, Roudot‐Thoraval, Lemerle, & Alvin, 2001). However, adherence may also depend on the type of referral received. In our recent Cochrane Review, for example, we found that treatment completion rates may be higher in young people referred to Dialectical Behaviour Therapy for Adolescents (DBT‐A) or to family therapy as compared with treatment as usual (Witt et al., 2021a), although the selection of recipients for these treatments may have been a factor.

Since our Cochrane Review was published in 2021 we have updated our systematic search using the same search strategy on 16 December 2023, finding only one further evaluation of an intervention relevant to the prevention of repeat self‐harm in children and adolescents. In this trial, a novel 12‐week therapist‐guided internet‐delivered emotion regulation therapeutic approach in addition to treatment as usual (TAU) was compared with TAU alone (Bjureberg et al., 2023). This approach, which is designed to address emotion dysregulation by adapting principles of acceptance‐based behavioural treatment, was found to be effective in reducing both self‐ and other‐reported NSSI frequency at both the 1‐month and 3‐month post‐treatment assessments. Significant effects were also observed for a number of putative mechanisms of action, including global functioning, emotion dysregulation, borderline personality disorder symptoms and psychological distress. Several trials of varied psychosocial and other interventions are planned or currently underway, including CBT‐ (Chaudhry, 2023) and DBT‐ (Rodante, 2022) based approaches, emotion‐regulation (Pagsberg, 2021), mixed interventions combining different therapeutic approaches (Berk, 2023; Bjureberg, 2023; Braquehais, 2023), neuro‐ and bio‐feedback (Zetterqvist, 2022), neuromodulation therapy (Tian, 2023b), transcranial magnetic stimulation (Tian, 2023a; Wu, 2023) and ketamine (Falcone, 2021).

Given what has been discussed above, there is a clear need to identify and overcome the challenges in designing, implementing and evaluating treatments for the prevention of self‐harm and its repetition in children and adolescents in clinical settings. The aims of this review, therefore, are to both provide an overview of these challenges and to provide clear and practical recommendations for clinicians to facilitate the real‐world application of the current research evidence into practice.

### Challenges in designing clinical interventions for youth who self‐harm

There is rarely one single cause of youth self‐harm. Instead, it is often the result of a complex interplay of factors, including possibly genetic, biological, psychological, psychiatric, social, cultural and other influences (Hawton, Saunders, & O'Connor, 2012), along with specific precipitating problems (Townsend et al., 2022). This complexity means that one single treatment approach is unlikely to be applicable and effective for all young people at risk. As a consequence, a number of different therapeutic approaches have been developed to try to improve psychosocial outcomes in young people, and to prevent repeat self‐harm and suicide. Whilst there is some evidence of a lower rate of self‐harm repetition for children and adolescents receiving DBT‐A (30%) compared with those in a control group (43%) at post‐intervention (odds ratio [OR]: 0.46, 95% confidence interval [CI] 0.26 to 0.82), most other interventions developed to date, regardless of their specific therapeutic approach, are associated with similar effects for repetition of self‐harm as with comparator treatments (e.g. Cognitive Behavioural‐based Therapy [CBT]: 0.93, 95% CI 0.12 to 7.24; Mentalisation‐Based Therapy for Adolescents: 0.70, 95% CI 0.06 to 8.46) (Witt et al., 2021a). This suggests that current treatment approaches may not be adequately addressing the underlying psychological processes that drive self‐harm behaviour (Fox et al., 2020).

Relatively few longitudinal studies have investigated the factors associated with the onset of self‐harm in young people, and even fewer have investigated factors associated with frequently repeated self‐harm. Whilst interpersonal difficulties with family, romantic partners and/or peers (Tilton‐Weaver, Latina, & Marshall, 2023; Townsend et al., 2022), a negative attributional style and symptoms of depression (Barrocas, Giletta, Hankin, Prinstein, & Abela, 2015) have been longitudinally associated with repeat self‐harm in young people, the field still lacks a comprehensive theory to explain the maintenance of self‐harm during adolescence (Tilton‐Weaver et al., 2023). Enhanced knowledge of the factors that may drive repeated self‐harm in particular may also help better tailor treatments to specific subgroups of young people.

Some current interventions are complex and multi‐component and may have a relatively prolonged duration of treatment. For example, CBT‐based approaches have a typical duration of up to 15 weeks. DBT‐A has a typical duration of between approximately 3 to 6 months, and family therapy has a typical duration of 18 months. Many of these approaches are also highly time intensive. For example, DBT‐A typically involves weekly group, family and individual sessions, as well as telephone calls. There is also a large degree of overlap in terms of the active components of these various forms of therapy (Witt et al., 2021a). Recent reviews that have statistically combined results from trials of different treatments with different therapeutic aims and approaches, moreover, have set limits on our understanding of which component(s) are most effective for clinically relevant subgroups of young people (Iyengar et al., 2018; Kothgassner, Robinson, Goreis, Ougrin, & Plener, 2020; Yuan, Kwok, & Ougrin, 2019). This makes it difficult to identify which component(s) may be most effective. Despite this, a recent practitioner review identified several components that may be associated with improved treatment effectiveness, including relationship building, case conceptualisation, skills training and the involvement of families (Meza, Zullo, Vargas, Ougrin, & Asarnow, 2023). An important challenge is to design and evaluate interventions that are brief and scalable, with potential for delivery by non‐specialist or low‐intensity practitioners, in order to meet the need of young people who may not be offered high‐intensity interventions such as DBT.

Other factors that may be associated with an increased risk of self‐harm and suicide are typically not a focus of many current treatment approaches. For example, there is increasing recognition that self‐harm‐related mental imagery may be associated with a higher risk for self‐harm behaviour in some young people (Cloos, Di Simplicio, Hammerle, & Steil, 2020; Lawrence et al., 2021). A recent review involving studies across the age range, for example, found that the majority of those presenting to clinical services report such imagery prior to their self‐harm episode (Lawrence, Balkind, Ji, Burke, & Liu, 2023). Therapeutic approaches addressing such imagery, as well as encouraging mental imagery associated with positive emotions, might help people develop alternative behaviours to self‐harm (Lawrence et al., 2023). A small feasibility study of electronically delivered functional imagery training has shown a beneficial effect for this approach on self‐reported self‐harm frequency at 3 months post‐intervention (Di Simplicio et al., 2020). This approach therefore clearly warrants further evaluation using more robust study designs.

The majority of the interventions trialled to date have been developed for adults and only minimally adapted, if at all, for young people. This is particularly so for CBT‐based approaches. Whilst there is increasing evidence of greater use of co‐design in the development of digital interventions for young people (Jones et al., 2020), there is little evidence of meaningful co‐design of face‐to‐face interventions for young people at risk of self‐harm and/or suicide. Of the 17 trials included in our recent Cochrane Review, for example, none reported that the intervention component(s), implementation, and/or evaluation strategies were co‐designed with young people and/or their carers or families (Witt et al., 2021a). Meaningful engagement of young people at all these stages is critical if we are to redesign youth mental health services for self‐harm in ways that improve treatment attendance, adherence and ultimately, outcomes for young people (Malla, Boksa, & Jooper, 2021). But there are challenges. Greater funding and time are often required to iteratively design the intervention (McCabe et al., 2022); more than is typically available to researchers under current funding models in many countries. For example, in one study the researchers found that to develop what was to be a relatively low‐intensity short message service‐based remote contact intervention, the iterative co‐design process took longer than anticipated, and resulted in an approach that was far more complex than anticipated. This, in turn, had implications for the feasibility of the planned pilot trial, from both funding and resourcing perspectives (Owens et al., 2011).

Project staff may also lack the experience and structural support needed to safely involve young people in the co‐design of research (Viksveen et al., 2022; Wadman, Williams, Brown, & Nielsen, 2019). In turn, human research ethics committees may be hesitant to approve such activities, particularly where co‐design activities involve young people with lived and living experience of self‐harm where safety considerations are paramount. To help professionals navigate some of these challenges, guidelines have recently been released to assist with involving young people with lived and living experience of self‐harm in co‐design (Webb et al., 2023). These provide suggestions for how researchers can create a safe environment for young people by, for example, undertaking training in managing distress, difficult conversations and group dynamics. There are also recommendations on creating clear and actionable safety and well‐being plans should distress arise as a result of co‐design activities. Suggestions for evaluating the involvement of young people in these activities in an iterative and ongoing manner to improve practice are also provided.

### Challenges in implementing clinical interventions for youth self‐harm

Our Cochrane Review found that around half of young people who engage in self‐harm and are offered therapy did not attend all planned sessions in the acute phase of treatment (excluding any booster sessions, if relevant), irrespective of the therapeutic approach (Witt et al., 2021a). Given that RCTs are often resourced more intensively, and emphasis is placed on engaging participants as much as possible, it is likely that rates of treatment non‐adherence are even higher in routine clinical settings (Granboulan et al., 2001). There is therefore a need to ensure that interventions are implemented in a way that is engaging for young people. For example, some work has suggested that greater family involvement may help to improve treatment engagement rates (Clarke, Allerhand, & Berk, 2019). However, such involvement and the extent of it needs to be carefully considered, taking into account any systemic issues. Discussion with the young person beforehand is essential.

Many clinicians and researchers have looked to telehealth, and particularly digital and online interventions, to develop more engaging interventions for young people who self‐harm. These interventions may either be offered as self‐guided interventions (e.g. Franklin et al., 2016), or alternatively, as an adjunct to traditional face‐to‐face therapy (e.g. Bjureberg et al., 2023). The growth of digital and online interventions for mental health has also been accelerated by the stay‐at‐home orders issued across many countries as a result of the COVID‐19 pandemic. For example, in Australia even though face‐to‐face sessions became acceptable again following the end of the acute phase of the pandemic, across the age range, one in five mental health treatment sessions now occur via telehealth as compared with only 5% in 2019 (Australian Institute of Health and Welfare, 2023). There is some suggestion that the proportion of treatment sessions occurring via telehealth as compared with face‐to‐face after the COVID‐19 lockdowns may, in fact, be somewhat higher in adults as compared to young people (84.5% vs. 66.0%) (Hoffnung et al., 2021). Whilst these technologies have the ability to increase access to care, they may not be equally appropriate for every person. Whilst many young people may prefer telehealth consultations to face‐to‐face appointments, it may not be appropriate for very young children, those unknown to the service, those with physical or behavioural concerns (Gormley, Melia, Mccormack, Phayer, & Madden, 2023), or those with non‐verbal autism‐spectrum diagnoses (Fell, Albright, Kryszak, Butter, & Kuhlthau, 2023). For young people experiencing cyberbullying, moreover, it remains unclear at present whether this would exacerbate their feelings of distress. Digital and online interventions may also perpetuate health inequalities as those without the knowledge required to access these services and/or without stable and reliable internet connectivity due to personal cost and/or provision limitations in their area may not be able to benefit (Gormley et al., 2023; Worsley, Hassan, Nolan, & Corcoran, 2022). There are also important safety implications. Therapists may not be able to assess non‐verbal cues (Worsley et al., 2022), or see that a young person has engaged in recent self‐harm. Reliance on digital, online, and/or telehealth services alone for young patients who have self‐harmed is therefore not recommended at the present time. Additionally, the evidence‐base both for self‐guided interventions and those designed to serve as an adjunct to traditional face‐to‐face therapy remains poor at present for this population, with few studies having rigorously evaluated these interventions to date (Witt, 2017).

### Challenges in evaluating clinical interventions for youth self‐harm

Some have questioned whether RCTs represent the best form of evidence for this field (Hawton & Pirkis, 2017). The RCT methodology was principally designed to evaluate pharmacological therapies that are not intended or expected to be modified frequently, and which have relatively predictable effects on the outcome(s) of interest (Mohr et al., 2015). Psychological interventions, on the other hand, can vary significantly as a result of resourcing, therapist experience and training and fidelity to the intervention manual, among other factors. Their effects are therefore not predictable, even from one RCT to another, let alone once rolled out into routine clinical practice.

Many treatment reviews, including our own, have also concluded that a number of interventions for self‐harm in young people appear to be equally effective (or ineffective) compared to the control or comparator condition (Kothgassner et al., 2020; Morken, Dahlgren, Lunde, & Toven, 2022; Witt et al., 2021a). In research on psychological interventions, the therapy chosen for inclusion in the control condition can vary considerably between studies. On the one hand, it may often be complex, multicomponent (due to ethical considerations precluding the use of a control condition without an active component with this patient population) and likely to have various effects on the outcome(s). With time, moreover, many components of the experimental treatment are likely to have become embedded into the treatment as usual (i.e. control) arm as services have improved over time in response to the evidence. This makes the choice of what is an appropriate control condition in these trials potentially very influential. We have previously shown, for example, that there is nothing ‘usual’ about treatment as usual. Instead, outcomes for CBT‐based psychotherapy for self‐harm can vary by as much as 2.7‐fold depending on the content of the treatment as usual condition (Witt et al., 2018). On the other hand, pragmatically, the control conditions of some studies may be much less targeted or specific; for example, an outpatient appointment to address any underlying/concurrent issues or a school counsellor appointment. It is important to evaluate whether an intervention is more useful than a minimal control intervention of this sort, given that for some young people, self‐harm is self‐limiting and may not warrant a specific intervention. Greater consideration should be given to the selection of comparator conditions in trials of psychological interventions in future trials, such as considering the appropriateness of the control condition and any potential interactions with the experimental intervention condition, controlling threats to internal validity and monitoring fidelity, therapist selection procedures and allegiance effects equally between the intervention and control conditions (Mohr et al., 2009). Clear reporting of the component(s) included in the control condition is also essential (Witt et al., 2018).

The estimated incidence of repeat self‐harm is relatively low even in high‐risk populations comprising those who have presented to the hospital on at least one prior occasion for self‐harm. In one Australian study, for example, the incidence of repeat self‐harm within 1 year of an index presentation was 188.8 per 1,000 person‐years. The incidence of suicide over this same period was around 3.3 per 1,000 person‐years (Qian et al., 2023). Given this, many trials are still underpowered to detect significant effects for self‐harm repetition and certainly for suicide. Using data from our recent Cochrane Review, we estimate that trials need to recruit between 200 (based on results for repetition of self‐harm for DBT‐A trials) to 57,670 (based on results for repetition of self‐harm for CBT‐A trials) young people per arm in order to detect a significant effect for repetition of self‐harm at post‐intervention with 80% power at the conventional alpha level. To evaluate impact on a suicide endpoint would likely require many more participants in each trial arm.

Few trials include outcomes of most importance to children and adolescents. Whilst most trials evaluate behavioural outcomes, such as absolute reduction in repetition of self‐harm, young people instead hope that treatment will lead to improved coping skills, greater acceptance, greater ability to speak safely about their self‐harm with others, and increased interest in activities they previously enjoyed. Reduction in the frequency of self‐harm (but, notably, not absolute cessation) is rated of lesser importance (Knowles et al., 2022).

### Where to from here? Addressing the challenges in research

Given the challenges identified in the previous sections, there is a need to design, implement and evaluate interventions taking these into account. Greater research use of newer ambulatory assessment approaches, such as ecological momentary assessment, which aims to investigate the sequence of events preceding self‐harm in real‐time and real‐world settings, may help to improve the identification of proximal risk factors for self‐harm, leading to the development of new treatment approaches. Whilst researchers have also suggested that these methods enable individualised interventions to be delivered according to self‐reported risk, others have highlighted the potential limitations to scalability of such a highly individualised approach (Kleiman, Glenn, & Liu, 2019).

There is also a need to identify the treatment component(s) associated with the greatest effectiveness to reduce, if possible, the duration of these often relatively prolonged and multicomponent treatments. A recent practitioner review, discussed earlier, identified several common elements of effective treatments for youth self‐harm based on a vote‐counting method and after combining studies, irrespective of therapeutic approach (Meza et al., 2023). Using appropriate methods to further articulate the potential mechanism(s) of action of these treatments is likely to also improve treatment adherence, which is known to be lower for young people compared with adults.

Novel treatment approaches are also required. Given previous studies on the role of self‐harm‐related mental imagery in those with suicidal thoughts and behaviour (Holmes, Crane, Fennell, & Williams, 2007; Lawrence et al., 2023), future studies could include treatment components to manage self‐harm‐related mental imagery. Pilot evaluations of such approaches appear to show promise in reducing the frequency of self‐harm up to 6 months post‐intervention in both community and clinical samples of young people (Di Simplicio et al., 2020).

In evaluating treatment approaches for the prevention of youth self‐harm, consideration should be given to alternative research designs, such as pragmatic clinical trials, adaptive clinical trials (particularly those that make use of leapfrog or Sequential, Multiple Assignment, Randomised Trial [SMART] designs) and registry‐based randomised controlled trials (RRCTs). By leveraging routinely collected electronic health records or data from administrative datasets to identify eligible participants and to collect information on the type(s) of treatment received, as well as outcome information (e.g. repetition of self‐harm or suicide), these designs are suited to testing hypotheses related to the evaluation of existing interventions, particularly where there is uncertainty about the best treatment combination, sequence or duration, or where multiple treatment options exist. Furthermore, as data linkage and quality improve, these designs can be used to assess clinical questions in a pragmatic and cost‐effective manner. By utilising registries as the foundation for such studies, these designs also offer advantages over traditional RCT approaches, such as facilitating rapid recruitment, increased statistical power and better generalisability to real‐world clinical settings. Greater use of these designs, and particularly RRCTs, is also likely to assist with clarifying the treatment component(s) received by young people assigned to the comparator condition as the same level of detailed information on treatment received, dose, duration and adherence would be recorded in a quality clinical register, irrespective of whether a young person was allocated to the treatment or comparator condition.

Consideration should also be given to methods to improve treatment adherence. For example, some work has suggested that the addition of gamified elements could help to ensure digital interventions remain engaging for young people in the longer‐term (Franklin et al., 2016). Involving the family has also been shown to improve adherence with some young people (Clarke et al., 2019), although as noted above this must be done judiciously.

## Conclusions and recommendations for practitioners

Notwithstanding the above challenges in research, clinicians need to apply the current research evidence to their practice if we are to realise much‐needed reductions in base rates of self‐harm and suicide in young people. To this end, we outline some basic principles of best practice to guide clinicians in this respect:

1. A comprehensive therapeutic assessment following self‐harm should be undertaken after each episode. Not only could this help to increase engagement with subsequent treatment, particularly if that treatment is provided in a timely fashion, but these assessments also offer the opportunity to develop a formulation that clarifies what needs to be addressed in treatment (Meza et al., 2023), including concurrent depressive and eating disorders (Warne et al., 2021), as well as contributory factors to self‐harm such as harmful or unhelpful internet and/or social media use (Susi, Glover‐Ford, Stewart, Knowles Bevis, & Hawton, 2023). A therapeutic assessment should also incorporate approaches that may reduce future risk, such as safety planning, which has shown promise in adults (Nuij et al., 2021), rather than a focus on risk prediction per se (Hawton, Lascelles, Pitman, Gilbert, & Silverman, 2022).
2. In both training and clinical practice there should be emphasis on the importance of establishing an empathic therapeutic relationship during initial assessment and subsequent interventions. Models such as the Collaborative Assessment and Management of Suicidality (Jobes, 2012) and Therapeutic Assessment (Tharinger et al., 2009) have both been found to improve engagement and cooperation between therapists and young people.
3. DBT‐A shows the most promise for reducing both the absolute repetition of self‐harm and, to a lesser extent, also the frequency of repeated self‐harm in young people. However, DBT‐A is a relatively prolonged and intensive form of psychotherapy that is unlikely to be appropriate for most young people who self‐harm.
4. Given the evidence of benefit for CBT in adults, it is possible that a modified version of this approach may also have benefits for some young people, although currently, evidence is lacking (Witt et al., 2021a). Specific adaptations that need to be made include modifying the language to suit the younger age group and involving family members as appropriate. It is important to consider including specific self‐harm and suicidal‐related content in the treatment for depression in young people given that self‐harm ideation may persist even when depression is treated (Witt, Madsen, et al., 2021). There is a pressing need to design these interventons in partnership with young people to enhance their acceptability and, potentially, their effectiveness.
5. Greater family involvement in treatment may reduce non‐adherence and improve treatment outcomes (Clarke et al., 2019). The extent of family involvement needs to be carefully tailored to the context, taking into account the views of the young person.
6. Most evidence‐based psychosocial approaches trialled to date have a relatively prolonged duration of treatment, are highly time intensive, and require access to highly experienced clinicians. These approaches are therefore ill‐suited to lower resource settings (e.g. lower income countries). There is some evidence that, in adults at least, contact‐based interventions such as regular follow‐up telephone‐contact over the post‐discharge period may be effective in these settings (Witt et al., 2021b). In settings where time is limited (e.g. emergency departments), safety‐planning interventions may be effective in reducing suicidal behaviour post‐discharge (Nuij et al., 2021). Brief, single‐encounter interventions (e.g. safety planning, care coordination) may also be effective in these settings (Doupnik et al., 2020). Further research is needed to evaluate these less intensive interventions in young people particularly.
7. To date, there have been no published trials of pharmacological agents specifically for the prevention of self‐harm and/or suicide in young people. Even in adult populations, the pharmacological agents tested to date are relatively old, often no longer in routine clinical use and do not show evidence of benefit for self‐harm or suicide endpoints (Witt et al., 2021a). Therefore, outside of their indication for specific psychiatric disorders (e.g. antidepressants for concurrent depression), we do not recommend the use of pharmacotherapy solely for the prevention of self‐harm or suicide in young people.
8. Given that negative experiences of clinical services may perpetuate a cycle of self‐harm (Uddin et al., 2023), consideration should be given to improving staff training to ensure all clinical staff feel equipped to treat the young people in their care in a person‐centred and compassionate manner. Providing easy access to immediate mentoring and supervision of ED staff who are involved with young people presenting after self‐harm should be considered (Veresova et al., 2024). This may be particularly important for staff working in emergency departments (Byrne et al., 2021) and in general hospitals (Uddin et al., 2023) who may encounter young people in crisis. Consideration should also be given to dedicating a separate area within the ED to triage and assess these presentations (Veresova et al., 2024).

**Key points**

- This review highlights the considerable challenges in designing, implementing and evaluating novel clinical interventions for young people who self‐harm.
- There is a need for further research to address these challenges, collaborating with young people at each stage of the development, implementation and evaluation process.
- A comprehensive assessment should be undertaken by a mental health professional after any episode of self‐harm resulting in presentation to a general hospital. This should include a formulation of factors contributing to the self‐harm and a risk management plan.
- DBT‐A shows the most promise as an intervention for self‐harm in young people but is unlikely to be realistic for most young people.
- CBT adapted to the adolescent context may be of benefit for some young people.
- Paying attention to the therapeutic relationship and involving the family wherever possible are important components of any therapeutic modality.
- Pharmacotherapy is not recommended solely for the prevention of self‐harm in young people.
- Attitudes of professionals in clinical services may influence the outcome of young people who self‐harm and are an important consideration in training.

## Data Availability

All data are reported in the original Cochrane Review cited within.
